# Treatment of affective dysregulation in ADHD with guanfacine: study protocol

**DOI:** 10.3389/frcha.2025.1547672

**Published:** 2025-03-28

**Authors:** Johanna Waltereit, Anne Uhlmann, Christos Tarassidis, Ulrich Preuss, Veit Roessner, Robert Waltereit

**Affiliations:** ^1^ Department of Child and Adolescent Psychiatry, LWL-Klinikum Marsberg, Marsberg, Germany; ^2^ Department of Child and Adolescent Psychiatry, Medical Faculty, German Center for Child and Adolescent Health (DZKJ), Dresden, Germany; ^3^ Department of Child and Adolescent Psychiatry, Klinikum Lippe, Bad Salzuflen, Germany; ^4^ Department of Child and Adolescent Psychiatry, University Medical Center Göttingen, Göttingen, Germany; ^5^ Department of Psychiatry, Psychotherapy and Preventive Medicine, Ruhr University Bochum, Bochum, Germany

**Keywords:** ADHD, oppositional-defiant disorder, conduct disorder, affective dyregulation, emotional dysregulation, guanfacine

## Abstract

The early onset, high prevalence, persistence and comorbidity of developmental disorders make affective dysregulation (AD) in childhood and adolescence one of the conditions with the greatest psychosocial burden and economic impact on society. Despite ongoing research, there remains a substantial need to optimize individualized treatment strategies to improve treatment outcomes and alleviate subjective distress and economic costs. The objective of this study is to investigate psychopathological markers of AD in children and adolescents with attention-deficit/hyperactivity disorder (ADHD) treated with the drug guanfacine as part of routine clinical care. These ADHD patients typically have not responded adequately to methylphenidate or other stimulant treatment. This study will employ a multicenter, single-arm design to evaluate the effects of guanfacine on AD symptoms in *n* = 40 patients of children and adolescents receiving regular ADHD treatment. The findings of this study are expected to address the hypothesis that guanfacine provides beneficial effects on symptoms of AD in addition to its effects on symptoms of ADHD.

**Public clinical trial registry:**

Affective Dysregulation (AD) in Children With ADHD Treated by Guanfacin, ClinicalTrials.gov ID NCT04016207.

## Introduction

### Scientific background

The terms “affective dysregulation (AD)” and “irritability” are often used interchangeably in the international literature. Most definitions of AD characterize it as a state of excessive reactivity to negative emotional stimuli, comprising both an affective component (anger) and a behavioral component (aggression). This means that individuals with AD tend to respond to provocations with exaggerated anger and aggressive behavior. AD or irritability is a criterion for many DSM-5 and ICD-10 diagnoses in children and adults, including mood and anxiety disorders, attention-deficit/hyperactivity disorder (ADHD), and oppositional-defiant disorder/conduct disorder (ODD/CD) (1).

Symptoms of AD are most prominently observed in ODD/CD (2, 3). Approximately 6% of adolescents (11–19 years) reported significant affective lability and 5.5% of parents reported that their children (8–19 years) exhibit frequent, rapid mood swings and irritability (4). The full diagnostic criteria of DSM-5 “disruptive mood dysregulation disorder” (DMDD) are met by 0.8% to 3.3% of children and adolescents. However, the prevalence of severe anger outbursts and chronic negative mood irritability is significantly higher, often alongside with comorbid psychiatric disorders (5).

Population-based studies indicate that the prevalence of disruptive behavior disorders ranges from 14% to 35% in children with ADHD, 14%–62% in children with anxiety disorders, and 9% to 45% in children with mood disorders (6). Moreover, children and adolescents with ODD/CD often exhibit symptoms of post-traumatic stress disorder at elevated rates. Children and adolescents with externalizing behavior disorders report lower levels of psychosocial well-being compared to their peers in the general population (7).

From both clinical and scientific perspectives, the AD concept fits well within the framework of the National Institute of Mental Health Research Domain Criteria (RDoC) Initiative (8). The RDoC approach focuses on dimensional constructs that span multiple diagnostic levels and can be examined in and across different areas and interfaces. Notably, the current RDoC includes the construct of frustration due to lack of reward within the broader domain of negative emotionality.

Guanfacine is an approved pharmacological treatment for ADHD in Germany. It is approved for patients aged 6–17 years as a second-line medication when methylphenidate or other stimulants are ineffective or poorly tolerated.

Guanfacine is a selective alpha-2A receptor agonist (9) with pharmacological properties similar to those of clonidine. Clonidine, a well-established antihypertensive medication, acts on alpha-2 receptors in the brain, thereby reducing peripheral vascular resistance, which results in lower blood pressure. In the United States, unlike in Germany, clonidine is also approved for the treatment of ADHD. Its therapeutic effect in ADHD is thought to arise from an increase in noradrenergic tone in the prefrontal cortex (PFC), facilitated through direct binding to postsynaptic alpha-2A adrenergic receptors and indirect increase of norepinephrine input from the locus coeruleus (10). Guanfacine exerts its effects by selectively activating alpha-2A adrenoceptors in the central nervous system, leading to reduced peripheral sympathetic nervous system activity, as evidenced by decreases in both systolic and diastolic blood pressure (11). In ADHD, guanfacine enhances attentional regulation and behavioral control via its action on the prefrontal cortex. These strengthening effects on prefrontal cortical functions are thought to result from inhibition of intracellular cAMP signaling (12, 13).

Clonidine has been shown to reduce AD-like symptoms, such as hyperarousal or aversive inner tension in adults with borderline personality disorder (14, 15). AD represents a transdiagnostic dimension characterized by excessive reactivity to negative emotional stimuli, involving both an affective (anger) and a behavioral component (aggression). This construct is not limited to behavior in borderline personality disorder, but is also observed in ADHD and social behavior disorders. As described above, guanfacine shares significant pharmacological similarities with clonidine. However, unlike clonidine, guanfacine is approved in Germany for the treatment of ADHD in children and adolescents aged 6–17 years. These pharmacological and clinical parallels led us to hypothesize that guanfacine could reduce AD symptoms in ADHD.

In ADHD, treatment with stimulants, particularly methylphenidate, is highly effective in reducing the core symptoms of attention deficit, hyperactivity and impulsivity (16). Nevertheless, approximately 30% of patients with ADHD exhibit an inadequate response to monotherapy in clinical studies (17). A common explanation for this can be the comorbidity of ADHD and ODD/CD. In case of hyperkinetic conduct disorder, which describes this common comorbidity, methylphenidate usually treats the core symptoms of ADHD, while the ODD/CD component is less significantly changed [MTA (18)]. Effective treatment of ODD/CD often requires long-term psychosocial interventions with either behavioral treatment or support in educational processes in the family and at school (19).

The core symptoms of ODD/CD exhibit substantial overlap with the concept of AD. In severe cases, off-label psychopharmacological interventions are commonly employed in Germany, particularly given the limited efficacy of purely psychosocial long-term interventions. Despite this, there is currently no approved pharmacological treatment for this indication. Antipsychotics, such as risperidone, are often prescribed for managing severe ODD/CD (20). However, their use in children and adolescents has often been criticized due to well-documented long-term metabolic side effects (21).

### Research question

In this study, we hypothesize that patients with ADHD who demonstrate an inadequate response to methylphenidate often present with comorbid ODD/CD and, as such, exhibit elevated levels of AD. Given that guanfacine is an approved treatment for ADHD and that emerging evidence suggests its potential efficacy in managing AD, we further hypothesize that guanfacine may alleviate AD symptoms in an ADHD subpopulation that is refractory to methylphenidate and other stimulants.

To date, no studies have specifically investigated the direct effects of guanfacine on AD in patients with ADHD. However, studies in patients with autism spectrum disorder have reported reductions in oppositional-defiant symptoms following guanfacine treatment (22, 23). A meta-analysis of alpha-2 receptor agonists revealed a significant effect of guanfacine monotherapy on oppositional symptoms in ADHD. No significant effect was found for clonidine (24). For guanfacine, a mean effect size of 0.51 (Hedges’ g) was reported, based on a single study (25).

### Aims of the study

In clinical practice, treating physicians prescribe guanfacine to children and adolescents with ADHD who have not responded adequately to stimulant therapy. The primary aim of this study is to investigate whether AD was changed as a side effect (in the sense of an epidemiological observation), as measured before (T1) and after (T2) dosing, while the main effect of guanfacine is on ADHD—core symptoms. Four different measurement instruments will be used to assess AD:

1. DADYS (parent and self-report versions): This instrument is part of the ADOPT consortium's efforts to systematically investigate AD in children and adolescents. The DADYS screen is intended to assess AD in children and adolescents. So far, the tool has been validated in a sample of children 8–12 years old (26).
2. Conners Comprehensive Behavior Rating Scales–Third Edition (Conners-3): A widely recognized and validated instrument for assessing ADHD and its comorbidities, the Conners-3 explicitly evaluates symptoms of conduct and oppositional-defiant behaviors, which are closely related to affective dysregulation. Both parent and self-report versions are used.
3. Emotion Regulation Questionnaire for Children and Adolescents (ERQ-CA): Emotional instability, a construct that is developmentally related to affective dysregulation, is measured using this internationally established instrument. Although precursor symptoms of emotionally unstable personality disorder typically manifest later in adolescence, this tool is appropriate for the study's target age group of 6–17 years.
4. Child Behavior Checklist-Dysregulation Profile (CBCL-DP): Extracted from the CBCL-6–18R parent questionnaire, this measure assesses the domains of anxious/depressed mood, attention problems, and aggressive behavior. The CBCL-DP is an established and extensively validated tool for assessing dysregulation in children and adolescents, serving as a complement to the DADYS instrument, which is still undergoing evaluation in the ADOPT consortium.

In summary, the combined use of these four instruments allows for a comprehensive examination of AD directly as well as of two important clinical manifestations, ADHD in conjunction with ODD/CD and with emotional instability.

## Methods and analysis

### Study design

This is a non-interventional test in accordance with German Arzneimittelgesetz (AMG) §4(23). The design is multicentric, prospective, open, single-arm, longitudinal.

### Primary objective of the study

Change in DADYS-EF (Affective Dysregulation Questionnaire) between Visit 1 and Visit 2.

### Secondary objectives of the study

Changes between visits 1 and 2 in

• DADYS-KF.
• CBCL-DP.
• C3-L, child version, domains inattention, hyperactivity/impulsivity.
• C3-L, parent version, domains inattention, hyperactivity/impulsivity.
• C3-L, child version, oppositional behavior/aggression domains.
• C3-L, parent version, oppositional behavior/aggression domains.
• ERQ-CA.

### Number of patients

*N* = 40.

### Study population

The study population consists of children and adolescents aged 6–17 years with ADHD and clinically significant AD, who are treated with guanfacine by their treating physician for clinical indications and as part of regular clinical treatment. The observational study influences neither the diagnosis of ADHD nor the decision to and implementation of therapy with guanfacine. These processes remain entirely within the responsibility of the treating physician. The study team only recruits patients for observational purposes once the treatment decision has already been made.

Guanfacine is in Germany approved as second-line therapy for ADHD in cases where stimulant treatments has been ineffective or stimulants should not be prescribed. Clinical experience suggests that rates of AD are high among ADHD patients who do not respond adequately to stimulants. Given the regulatory approval of guanfacine for patients with ADHD aged 6–17 years, only children and adolescents within this age range are eligible for inclusion. While gender differences may occur, they are not anticipated to have a fundamental impact on study outcomes. Therefore, to ensure an adequate sample size and generalizability of findings, both boys and girls will be recruited.

### Inclusion criteria

• Male and female patients aged 6–17 years.
• Diagnoses F90.0 (ADHD) or F90.1 (hyperkinetic disorder of social behavior).
• Patients have not responded adequately to stimulants or these are not suitable for clinical reasons; switching to guanfacine is clinically indicated and regularly planned.
• Patients show clinical symptoms of affective dysregulation.
• IQ is at least 70.
• Written informed consent from the participating patients and their legal guardians.

### Exclusion criteria

• Unipolar depression, bipolar disorder, schizophrenia, or another psychotic disorder.
• Current substance abuse.

### Recruitment of patients

Children and adolescents diagnosed with ADHD who are receiving routine care at the outpatient clinic, day clinic or inpatient ward of the three study centers will be recruited: Department of Child and Adolescent Psychiatry, University Hospital Dresden, Department of Child and Adolescent Psychiatry, LWL-Klinikum Marsberg and Department of Child and Adolescent Psychiatry, Klinikum Lippe, Bad Salzuflen. In addition, practicing child and adolescent psychiatrists will be invited to identify eligible patients under their care who are planned to receive guanfacine as part of standard clinical treatment and who may be interested in participating in the study.

### Treatment with guanfacin as part of the regular clinical treatment (not part of the study)

Guanfacine treatment is conducted exclusively as part of routine clinical care in accordance with its regulatory approval and the current product information. In Germany, extended-release guanfacine “Intuniv” is approved. The treating physician retains sole responsibility for all aspects of patient care, including the diagnosis of ADHD, initiation of guanfacine therapy, obtaining informed consent, monitoring treatment response and side effects, ensuring patient safety, assessing clinical outcomes, and deciding on continued treatment. These processes are independent of the study team and explicitly not part of the observational study protocol. The following description of routine guanfacine treatment is provided for context to facilitate understanding the observational study protocol.

Guanfacine is administered exclusively as part of routine clinical care in child and adolescent psychiatry, independent of the observational study. It is regularly employed as a second-line treatment for patients with severe or treatment-resistant ADHD, as per its approval conditions. This practice is established both in Germany and at the participating study centers, and is completely independent of our observational study. The patients typically have a long history of treatment, as guanfacine is only approved for use in cases where there is a confirmed diagnosis of ADHD and an insufficient response to stimulants (methylphenidate, amphetamines), making it a second-line pharmacological treatment. These patients can be treated in outpatient, day-care or inpatient settings; their treatment history (patient is admitted to hospital from outpatient treatment for single-dose guanfacine) often includes a combination of these care levels. In other cases, practicing child and adolescent psychiatrists refer patients with ADHD to inpatient care for guanfacine dosing.

The decision to initiate guanfacine is solely made by the treating physician. However, in real practice, the decision does not lead to an immediate regulation, leaving time to assess study inclusion. Specifically, the decision is reviewed by the responsible senior physician, followed by laboratory testing, an ECG and, if necessary, a physical examination. The legal guardians will be provided with comprehensive information about the planned medication. This will include a detailed discussion of the potential effects, risks, and side effects, which will be documented. The entire process from treatment decision to guanfacine initiation typically spans 2–3 weeks. If patients and their legal guardians have previously consented to being contacted regarding research studies, the treating physician informs the study team about an eligible patient. At this point, the physician also assesses and documents whether clinically relevant symptoms of AD are present.

During the remaining interval prior to treatment initiation, the study team approaches the patient and their guardians to clarify interest and obtain consent for participation in the observational study. If consent is granted, the first measurement (T1) takes place before the start of guanfacine treatment. Importantly, the decision to use guanfacine and the entire treatment is solely under the responsibility and direction of the treating physician. Neither the inclusion in the study nor the results of the psychometric measurements (T1 and T2), which are not known to the treating physician, influence the guanfacine treatment or further treatment with guanfacine.

Guanfacine is administered according to the drug approval in Germany. The dosage in children and adolescents is up to 7 mg. During titration, an increase or decrease of 1 mg guanfacine per week is allowed.

**Figure 1:**
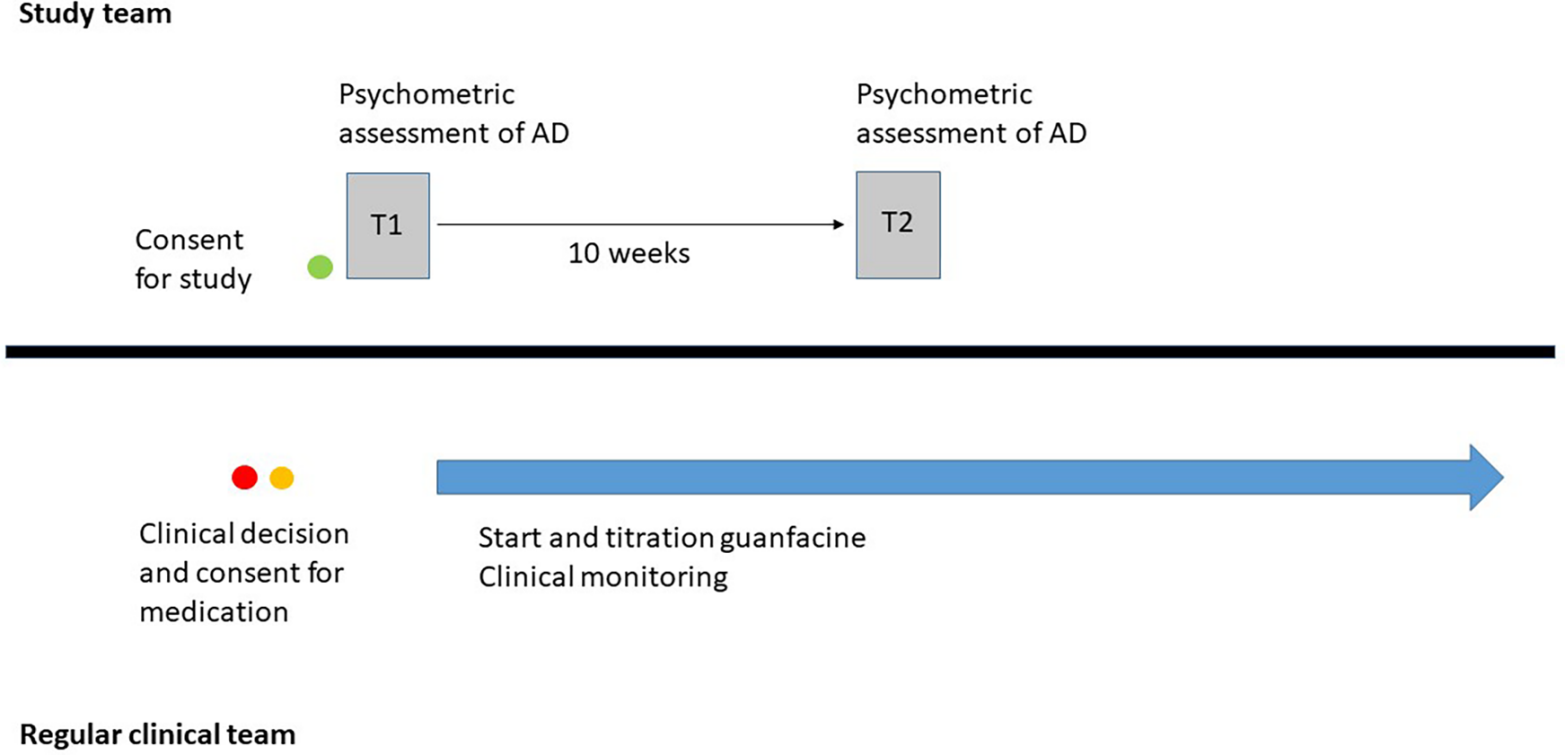
Overview of the study design.

Figure 1 illustrates the parallel processes of regular clinical treatment and observational study, whereby the observational study does not influence the regular clinical treatment.

### Study visit 1 (T1, day 1)

Prior to inclusion in the study, all patients and their guardians must receive comprehensive information about the study. This information will be provided personally by the treating doctor and in written form via patient information materials. Only after all questions from the patient and legal guardian have been fully clarified, will they be asked to sign and date two copies of the consent form (for patients and for legal guardians) by hand. One copy of the patient information/consent form will be handled to the participant, while the second copy will be retained in the study center's records.

During visit 1, demographic data and relevant clinical documentation, including the diagnosis and indication for guanfacine treatment, are recorded. Information regarding existing AD will be collected from the reports of the treating physician and used to eligibility for the study. Once the inclusion and exclusion criteria have been thoroughly evaluated, the participating person is included in the study. The intelligence quotient will be extracted from existing clinical records.

The following psychometric instruments are collected with the patient and a parent:

• DADYS-EF.
• DADYS-KF.
• C3-L, children's version.
• C3-L, parent version.
• ERQ-CA.

### Study visit 2 (T2, day 70 ± 2)

At Visit 2, the treating physician's documentation of guanfacine dosage will be recorded.

The following psychometric instruments will again be administered to the patient and a parent:

• DADYS-EF.
• DADYS-KF.
• C3-L, children's version.
• C3-L, parent version.
• ERQ-CA.

### Drop-outs

Participants can withdraw from the study at any time without providing a reason and without any consequences for their future treatment. The reason for withdrawal, if provided, is documented in the study records.

### Documentation

The study physician is responsible for ensuring that the study is conducted in accordance with the medical professional code, the Declaration of Helsinki and the study protocol, and that all data is correctly documented. All data collected during the study must be entered into the eCRF by authorized personnel, including data from participants excluded from the study.

The study center will maintain a patient identification list containing the participant number, full name, date of birth and date of inclusion. This list will remain at the study center upon study completion. In addition, the participation in the study including participant number, study start and end dates, will be documented in the medical record.

### Data management

All data entered by the study center will be managed and processed by the Clinical Trial Center (KKS) Dresden using the RedCAP software in accordance with data protection regulations. Should a study participant or their legal guardian requests data deletion, this will be carried out immediately.

The data will undergo range, validity and consistency checks. If necessary, queries wil be generated which authorized personnel will address. The study physician will review and resolve any discrepancies to ensure data accuracy. Upon completion of the study, the database will be closed only after all relevant data has been entered and queries resolved.

### Caseload planning

No prior studies in the literature have specifically examined the effect of guanfacine on AD in patients with ADHD. Therefore, there is no direct prior evidence regarding the effect of guanfacine on AD in ADHD patients. Indirect evidence comes from a meta-analysis reporting moderate effect sizes (Hedges’ g = 0.51) for guanfacine in reducing oppositional behaviors (24). Since the current study directly measures AD and targets a population with high baseline AD values due to non-response to stimulants, we assume medium to large effect sizes of Cohen's d = 0.65.

Sample size calculation was performed using G-Power program (http://www.gpower.hhu.de/). Assuning at least a medium effect sizes of Cohen's d = 0.50, a two-sided alpha error level of 5%, and a power of 0.90, a minimum of 34 participants is required. Accounting for a drop-out rate of approx. 15% the target sample size is *n* = 40 participants.

### Statistical methods

#### Outcome measures

Behavioral parameters will be assessed using self-report and parent-report rating scales at time point T1 (before guanfacine) and T2 (9 or 10 weeks after inclusion, guanfacine continued).

#### Population for analysis

The study population consist of participants measured longitudinally at two time points. Data from all *n* = 40 participants will be analyzed, including multiple imputation for missing data where necessary, and complete case analysis will be performed.

#### Data analysis

Differences between T1 and T2 will be analyzed using one-way analysis of variance (ANOVA), equivalent to a paired Student's *t*-test under normal distribution assumptions. Drop-out cases will not be included, but described in detail.

## Discussion

Based on clinical experience, we expect the results of this study to support our hypothesis.

To the best of our knowledge, this is the first study to investigate the effects of guanfacine on AD in ADHD. This strength helps to counterbalance potential limitations, such as the non-blinded, single-arm study design.

If the hypothesis is confirmed, the clinical implications for managing AD in children and adolescents could be substantial. Compared to antipsychotics, the primary pharmacological alternative, guanfacine is better tolerated, with a more favorable side-effect profile. Therefore, guanfacine would not only be an approved pharmacological treatment of AD in ADHD, but could also offer potential benefits in the treatment of AD independently of coexisting ADHD.

## Ethics and dissemination

### Need to conduct the clinical study

AD has a significant impact on the mental health of children, adolescents, and adults. In children and adolescents, the treatment of AD is a critical focus of psychotherapeutic and milieu-therapeutic interventions. However, in many cases, these approaches fail to achieve sufficient improvement, underscoring a significant need for additional treatment options, particularly pharmacological strategies. Currently, no pharmacological treatment is approved in Germany for the treatment of AD. Unofficial clinical case reports have suggested that guanfacine may be effective in the treatment of AD in children and adolescents with ADHD. As described above, there is supporting evidence in the literature indicating the potential efficacy of guanfacine in this context. Guanfacine is an already approved medication for treating the core symptoms of ADHD in children and adolescents. This study aims to address a critical gap by investigating, for the first time, whether guanfacine can also effectively reduce symptoms of affective dysregulation in addition to treating the core symptoms of ADHD. If successful, this study could offer significant advancements in the treatment of AD in children and adolescents and provide a much-needed pharmacological option for this challenging condition.

### Benefit-risk assessment

Ethical research projects must address new and relevant research questions while minimizing the burden on study participants in relation to the potential benefits. In studies involving children and adolescents, the need for special protection is a particularly important consideration. The study design described here has been carefully developed to ensure the research question is answered effectively while prioritizing participant safety and minimizing risks.

The research question—whether guanfacine has a beneficial effect on AD in an ADHD population—offers significant potential benefit. The question is novel and has not been answered despite preliminary indications from the existing literature. AD is associated with substantial distress in children and adolescents; and identifying of an additional treatment option for severe cases, particularly in the context of ADHD and potentially in other clinical constellations, would hold considerable clinical value.

Participation in the study involves no plausible or significant risks. The study will accompany regular clinical dosing of guanfacine in children and adolescents with ADHD by conducting psychometric assessments before and after treatment initiation. Data collection is limited to psychometric evaluations, completed by patients and their primary caregivers using standardized rating questionnaires. Consequently, the study imposes minimal burdens, as no study procedures are invasive in nature.

From a benefit-risk perspective, the potential benefits of this study clearly outweigh the risks. The study offers meaningful opportunities to gain clinically relevant insights while ensuring participant safety and minimizing inconvenience.

### Ethics statement

The study has been approved by the following ethics committees: Ethikkommission Dresden (EK 476102019), Ethikkommission der Universitätsmedizin Göttingen (22/3/21 Ü) and Ethikkommission der Ärztekammer Westfalen-Lippe (2023-368-b-S).
